# Comprehensive analysis of circRNA-miRNA-mRNA networks to reveal potential cell death, inflammation and oxidative stress-related targets for postoperative cognitive dysfunction

**DOI:** 10.3389/fnagi.2026.1733749

**Published:** 2026-09-11

**Authors:** Yajing Zhang, Shujun Lu, Ying Yu, Zhe Yan, Qingling Qi

**Affiliations:** 1 Department of Anesthesiology, First Teaching Hospital of Tianjin University of Traditional Chinese Medicine, Tianjin, China; 2 National Clinical Research Center for Chinese Medicine, Tianjin, China; 3 Department of Anesthesiology, Tianjin First Central Hospital, Tianjin, China

**Keywords:** circRNA-miRNA-mRNA, inflammation, oxidative stress, postoperative cognitive dysfunction, programmed cell death

## Abstract

**Background:**

Postoperative cognitive dysfunction (POCD) is a common neurological complication involving the hippocampus, a brain region critical for learning and memory. Circular RNA (circRNA) plays an important role in regulating gene expression. Our objective is to predict and verify a circRNA-miRNA-mRNA network in POCD.

**Methods:**

Microarray data of POCD mouse models were retrieved from the Gene Expression Omnibus (GEO) database. Differential expression analysis was used to identify differentially expressed circRNA, miRNA, and mRNA. An in silico circRNA-miRNA-mRNA network was subsequently predicted based on miRNA-mRNA pairs and miRNA-circRNA pairs obtained from public databases. From this global network, three hub subnetworks based on the competing endogenous RNA (ceRNA) hypothesis were analyzed. In hippocampal tissue of POCD mice, RT-qPCR quantified key circRNAs, miRNAs, and mRNAs, with protein levels of the corresponding mRNA confirmed by western blot. Dual-luciferase assays in HT22 cells verified interactions between mmu_circ_0001838 (mmu_circRNA_009748) and mmu-miR-337-3p, and between mmu-miR-337-3p and PLA2G12B mRNA.

**Results:**

Analysis of publicly datasets related to POCD identified 12 genes potentially associated with programmed cell death, inflammation, immunity, and oxidative stress in POCD. According to these results, we successfully predicted a circRNA-miRNA-mRNA network, comprising 4 circRNAs, 14 miRNAs and 12 mRNAs. Furthermore, three circRNA-miRNA-mRNA subnetworks were then derived based on the ceRNA hypothesis, which included 3 circRNAs, 4 miRNAs and 8 mRNAs: mmu_circRNA_016232-mmu_miR-6912-5p-four mRNAs (GBP2, BCL3, ADGRL4, PLN), mmu_circRNA_009748-mmu_miR-362-3p/mmu_miR-337-3p-two mRNAs (RSAD2, PLA2G12B), and mmu_circRNA_009396-mmu_miR-7030-5p-two mRNAs (SLC25A13, GPR171). The AUC value of each predicted circRNA-miRNA-mRNA path in diagnosing POCD exceeded 0.8. RT-qPCR results validated that the levels of mmu_circRNA_016232, mmu_circRNA_009748, mmu_miR-7030-5p, GBP2 mRNA, BCL3 mRNA, ADGRL4 mRNA, PLN mRNA, RSAD2 mRNA and PLA2G12B mRNA were elevated, while the levels of mmu_circRNA_009396, mmu_miR-6912-5p, mmu_miR-362-3p, mmu_miR-337-3p, SLC25A13 mRNA, and GPR171 mRNA were reduced in the hippocampal tissues of POCD mice compared to the control group. Western blot corroborated the protein-level changes for the corresponding mRNAs. Dual-luciferase assays further confirmed the direct interactions of mmu_circ_0001838 with mmu-miR-337-3p and mmu-miR-337-3p with PLA2G12B mRNA.

**Conclusion:**

This study predicted and verified three critical circRNA-miRNA-mRNA regulatory axes, providing new insights into the molecular mechanisms underlying POCD and identifying potential biomarkers for its diagnosis and intervention strategies.

## 1 Introduction

Postoperative cognitive dysfunction (POCD) is a common postoperative complication characterized by progressive memory impairment and cognitive decline (Newman et al., 2007). This condition is often accompanied by negative effects on mood regulation and personality traits (Le et al., 2014). POCD typically occurs following major surgical interventions (Evered and Silbert, 2018), particularly among the elderly population, with an incidence rate ranging from 15 to 60% (Urits et al., 2019). The consequences of POCD include prolonged hospital stays and a substantial decrement in the patient’s quality of life (Blokzijl et al., 2021), which can increase mortality risk in severe cases (Rundshagen, 2014). Furthermore, cognitive impairments associated with POCD may persist for protracted periods, spanning years, thereby augmenting the vulnerability to Alzheimer’s disease (AD) development (Lin et al., 2020). Therefore, the prompt assessment and diagnosis of POCD holds paramount significance in ameliorating patients’ quality of life and enhancing survival outcomes. However, the intricate pathophysiological mechanisms underlying POCD remain largely elusive, impeding the development of comprehensive preventive measures. The hippocampus, a key brain region responsible for learning, memory, and emotional regulation, plays a central role in the onset and development of POCD (Lei et al., 2024; Kalb et al., 2013). Extensive evidence indicates that hippocampal neuronal loss and atrophy, along with neuroinflammatory responses, are closely associated with cognitive decline (Agrawal et al., 2025; Pan et al., 2025; Chen et al., 2019). Therefore, in-depth analysis of molecular changes in the hippocampus is of great significance for elucidating the pathogenesis of POCD.

Numerous studies have demonstrated the intricate involvement of POCD in neuronal death, encompassing programmed cell death pathways, neuroinflammation within the central nervous system (CNS), and neurodegeneration induced by oxidative stress (Lin et al., 2020; Safavynia and Goldstein, 2018; Wang S. et al., 2024). Consequently, numerous investigations have identified potential therapeutic targets aimed at mitigating these pathological processes. For instance, Xue et al. revealed that *TREM2* activation reduces neuroinflammation, thereby ameliorating postoperative cognitive dysfunction (Han et al., 2022). Aging-induced down-regulation of Nrf2 in the hippocampus impairs the defense system’s ability to counteract surgical insults, rendering it more susceptible to oxidative stress and neuroinflammation, which contribute to POCD development (Li et al., 2023). Notably, MEF2C mitigates cognitive dysfunction following surgery by inhibiting ferroptosis, a type of programmed cell death (Wang S. et al., 2024). Additionally, HDAC6 in the hippocampus facilitates POCD by enhancing microglial pyroptosis induced by NLRP3 in aged mice (Lin et al., 2024). Thus, strategies aimed at inhibiting programmed cell death, reducing neuroinflammatory responses, and mitigating oxidative stress may potentially lead to improved clinical outcomes for patients suffering from POCD.

Circular RNAs (circRNAs) represent a category of non-coding RNAs characterized by distinctive features, such as a closed loop structure (typically ranging from ~100 to several thousand nucleotides in length) along with high levels of conservation and stability (Zhou et al., 2020). Recent research has highlighted the essential role that circRNAs play in the pathology of POCD, suggesting that they may act as crucial regulators in the development and progression of this condition (Wang X. et al., 2024; Zhang et al., 2022). For instance, circRNA001372 has been shown to reduce neurotoxicity and neuroinflammation via the PI3K/Akt/NF-κB signaling pathway in rat brain and nerve cells (Wang et al., 2021). Additionally, circRNAs are prevalent throughout the eukaryotic transcriptome and can function as miRNA sponges through the competitive endogenous RNA (ceRNA) mechanism, thereby relieving the inhibitory effect of miRNAs on their target mRNAs (Guo et al., 2022). Wang X. et al. (2024) reported that circAKT3 mitigates postoperative cognitive dysfunction in aged mice through targeting miR-106a-5p/HDAC4/MEF2C axis. Wen et al. (2025) revealed that circHOMER1 suppresses hippocampal neuronal injury induced by sevoflurane via targeting miR-217. Therefore, in-depth exploration of the circRNA-miRNA-mRNA regulatory network is of great significance for systematically clarifying the molecular mechanisms underlying POCD and identifying new targets for intervention.

In this study, we employed an integrative bioinformatics approach by combining POCD-related public datasets with public miRNA-target interaction databases to predict a comprehensive circRNA-miRNA-mRNA regulatory network associated with inflammation, oxidative stress, and programmed cell death in the hippocampus of POCD. We further investigated the primary biological functions and signaling pathways of the key target genes, aiming to provide new insights into the pathological mechanisms and potential therapeutic targets for POCD.

## 2 Materials and methods

### 2.1 Subjects

All datasets were derived from mouse (*Mus musculus*) hippocampal tissues, a key brain region for cognitive function. Two mRNA expression profiles (GSE115440 and GSE215410), one miRNA expression profile (GSE95070), and two circRNA expression profiles (GSE165798 and GSE190880) were downloaded from the Gene Expression Omnibus (GEO)^1^ database. The details of them were shown in Table 1.

**Table 1:** Expression profile information.

Series	Type	Sample	Numbers (POCD/Control)	Citation(s)
GSE115440	mRNA	POCD of mouse	6 (3/3)	30770053; 35116043
GSE215410	mRNA	POCD of mouse	4 (2/2)	36437988
GSE95070	miRNA	POCD of mouse	10 (5/5)	-
GSE165798	circRNA	POCD of mouse	6 (3/3)	34483886; 39138637
GSE190880	circRNA	POCD of mouse	6 (3/3)	-

For the analysis of mRNA sequencing data, the datasets GSE115440 and GSE215410 were merged: GSE115440 (comprising 3 control and 3 POCD hippocampal samples) and GSE215410 (comprising 2 control and 2 POCD hippocampal samples). In total, 5 control and 5 POCD samples were included for subsequent differential expression analysis. For the analysis of circRNA sequencing data, data from GSE165798 and GSE190880 datasets were merged: GSE165798 (comprising 3 control and 3 POCD hippocampal samples) and GSE190880 (comprising 3 control and 3 POCD hippocampal samples). In total, 6 control and 6 POCD samples were included for subsequent circRNA differential expression analysis. Batch effect correction was performed on the merged dataset with the ComBat function (R package sva, version 3.50.0), using the dataset source as the batch variable while preserving inter-group differences.

Besides, programmed cell death including apoptosis, necroptosis, autophagy, pyroptosis, ferroptosis, and cuproptosis-related genes, inflammation-related, oxidative stress-related genes, immunogene, and transcription factor-related genes were obtained from the Genecards database.^2^

### 2.2 Differential expression analysis

The “limma” R package (version 3.56.2) was used to identify differentially expressed genes (DEGs), differentially expressed miRNAs (DEmiRNAs), and differentially expressed circRNAs (DEcircRNAs) (Ritchie et al., 2015; Yang et al., 2026; Chi et al., 2025). The selection criteria were |log_2_FC| > 0.5 and FDR < 0.05.

### 2.3 Functional enrichment analysis

The Gene Ontology (GO) terms [including biological process (BP), molecular function (MF), and cellular component (CC)] were conducted to provide gene annotation terms, and the Kyoto Encyclopedia of Genes and Genomes (KEGG) was conducted to analyze the enrichment pathways of genes. Both GO and KEGG were performed using the “clusterProfiler” R function package (version 4.8.3) (Yu et al., 2012; Xiong et al., 2026; Wang L. et al., 2022).

### 2.4 Protein–protein interaction analysis

The STRING database^3^ was used to explore protein–protein interactions (PPIs). Besides, the online database GeneMANIA^4^ was employed to look for genes sharing functions.

### 2.5 Prediction of a circRNA-miRNA-mRNA network

To predict the circRNA-miRNA-mRNA network, the regulatory relationships between miRNAs and mRNAs were obtained from five databases including miRWalk,^5^ miRTarBase,^6^ Targetscan8.0,^7^ miRDB6.0,^8^ and ENCORI.^9^ Besides, the regulatory relationships between miRNAs and circRNAs were obtained from the CircFunBase database^10^ and the ENCORI database. Subsequently, the circRNA-miRNA-mRNA network was predicted by combining the circRNA-miRNA pairs and the miRNA-mRNA pairs.

### 2.6 Transcription factor binding site prediction

The 1,000-bp sequence file located upstream of the gene’s start site was downloaded from the UCSC website.^11^ The transcription factor (TF) corresponding motif files were downloaded from the JASPAR database.^12^ The occurrence of TFs binding sequences within the upstream region of the gene’s promoter was practiced using the online tool FIMO.^13^ Finally, the Cistrome database^14^ was used to find binding peaks of genes and TFs.

### 2.7 Animal study

All animal experiments were conducted in accordance with the guidelines of the Tianjin Medical Experimental Animal Protection Center. The animal experimental protocol was approved by the Animal Ethics Committee of Yishengyuan Gene Technology (Tianjin) Co., Ltd. (Approval No. YSY-DWLL-2024534). Male C57BL/5 J mice (18 weeks old, SPF grade) were obtained from Beijing Huafukang Biotechnology Co., Ltd. All mice were kept under controlled environmental conditions (temperature: 22 ± 2 °C; humidity: 50 ± 10%) with a 12-h light/dark cycle. A total of 18 mice were randomly assigned to two groups: the Control group and the POCD group (*n* = 9). The POCD model was established through exposure to sevoflurane via inhalation, as previously outlined (Gao et al., 2024). Briefly, the mice were exposed to continuous inhalation of 3% sevoflurane mixed with pure oxygen for a duration of 2 h over the course of 3 consecutive days (Gao et al., 2024). Following this procedure, the mice were anesthetized using 1% isoflurane inhalation, after which they were sacrificed by cervical dislocation, and the hippocampal tissue was isolated.

### 2.8 Reverse transcription real-time quantitative polymerase chain reaction (RT-qPCR)

Total RNA was extracted from mouse hippocampal tissue using TRIZOL reagent (No. 15596018CN, ThermoFisher Scientific). For the quantification of miRNA expression, the All-in-One™ miRNA qRT-PCR Detection Kit 2.0 (QP113, Genecopoeia) was utilized following the manufacturer’s protocol. For the analysis of mRNA and circRNA expression, cDNA synthesis was performed using the M-MLV RT Premix kit (AG11706, Accurate Biology), followed by quantitative PCR (qPCR) using the SYBR Green premix kit (AG11739, Accurate Biology). The relative expression levels were calculated using the 2^-ΔΔCt^ method (Qian L. et al., 2023), with β-actin serving as the internal reference gene (Jiang et al., 2022; Gao et al., 2026). The primers of miRNAs, including mmu_miR-337-3p, mmu_miR-362-3p, mmu_miR-6912-5p and mmu_miR-7030-5p, were synthesised by Genecopoeia. The sequence data for circRNAs was sourced from the circBase database (Supplementary Tables S1, S2). In order to accurately identify circRNAs, we designed back-to-back (divergent) primers spanning the back-splice junction. The primers of mRNAs and circRNAs were listed in Table 2.

**Table 2:** Primer sequences for qPCR.

Genes	Forward Primer (5′-3′)	Reverse Primer (5′-3′)
Gbp2	CCTGGTTCTGCTTGACACTGAG	TGCTGGTTGATGGTTCCTATGC
Bcl3	GCTGCTGAACCTGCCTACTC	GCGTGTCTCCATCCTCATCC
Adgrl4	CACCAGGACCACGATTCACAAG	GCCAGCAATGATAGAGCAGACC
Rsad2	CTGTGAGCATAGTGAGCAATGG	TGTCGCAGGAGATAGCAAGAAT
Pln	CTCACTCGCTCGGCTATCAG	GCAGATCAGCAGCAGACATATC
Pla2g12b	CGCTGAAGGACCGCTACAAG	TGAGGTGTCAACTGGCAACTG
Gpr171	GCTCTGCTCCACATTCTCTTG	ATATGACCTCTGTCTGGCTGAG
Slc25A13	CGTGGAGGTGACTAAGGAGGA	CCGCTCAATGTCTGCTAAGGT
mmu_circRNA-009748	TGATGGACTGGGAACAAAGGAA	CGTGACTGCACAATGAACTTGA
mmu_circRNA_009396	ATGCTTGCGAGGAGTTGATTG	TGAAGACCATCTGGAAGACTGA
mmu_circRNA_016232	AAAGACTCGGTGCTTCGGAAA	AAGTGGTTCTGTCTGACTGTGA
β-actin	GTACTCTGTGTGGATCGGTGG	GCAGCTCAGTAACAGTCCG

### 2.9 Western blot

Protein samples were separated by SDS-PAGE and subsequently transferred onto polyvinylidene fluoride membranes. The membranes were then blocked with 5% non-fat milk, followed by overnight incubation at 4 °C with the following primary antibodies: anti-GBP2 (Proteintech Group, Rosemont, IL, USA; 11,854-1-AP), anti-BCL3 (Proteintech Group, Rosemont, IL, USA; 23,959-1-AP), anti-ADGRL4 (Affinity, Cincinnati, OH, USA; AF9063), anti-PLN (Proteintech Group, Rosemont, IL, USA; 15,873-1-AP), anti-RSAD2 (Proteintech Group, Rosemont, IL, USA; 28,089-1-AP), anti-PLA2G12B (Proteintech Group, Rosemont, IL, USA; 25,799-1-AP), anti-SLC25A13 (Thermo Fisher, Waltham, MA, USA; PA5-103678), anti-GPR171 (Thermo Fisher, Waltham, MA, USA; PA5-10206), and anti-GAPDH (Proteintech Group, Rosemont, IL, USA; 60,004-1-Ig). After that, the membranes were incubated with corresponding secondary antibodies for 1 h at room temperature. Protein bands were visualized using an enhanced chemiluminescence (ECL) substrate and detected with a chemiluminescence imaging system.

### 2.10 Cell culture and transfection

Mouse hippocampal neuronal HT22 cells were cultured in DMEM-H medium supplemented with 10% fetal bovine serum and 1% penicillin–streptomycin at 37 °C with 5% CO₂. Mmu-miR-337-3p agomir and antagomir, as well as mmu_circRNA_009748-overexpressing plasmid (circRNA-OE) and mmu_circRNA_009748-specific siRNAs (mmu_circRNA_009748 siRNA1, siRNA2 and siRNA3) were synthesized by Tsingke Biotechnology Co., Ltd. and transfected using the Lipofectamine 3,000 reagent. The sequences of mmu-miR-337-3p agomir and antagomir, mmu_circRNA_009748 siRNAs, and their respective negative controls (NC) were listed in Supplementary Table S2.

### 2.11 Dual luciferase reporter assay

HT22 cells were co-transfected with pmirGLO vectors (GENINK BIOSCIENCE, Tianjin, China) carrying wild-type or mutant mmu_circ_0001838 (also known as mmu_circRNA_009748) and carrying cDNA to the wild-type or mutant 3’ UTR of PLA2G12B mRNA, together with mmu-miR-337-3p mimics or NC using Lipofectamine2000. Luciferase activities were measured with the Dual-Luciferase Assay Kit (RG027, Beyotime) following the manufacturer’s protocol.

### 2.12 Statistical analysis

The Wilcox test was conducted to assess the differences between two groups. All the networks were visualized using the Cytoscape software (version 3.10.2). The “cor” function of R language was performed to analyze the Pearson correlation. All statistical analyses were conducted using R software (version 4.3.3). Student’s *t*-test was used to analyze comparisons between two groups; one-way ANOVA was used for comparison between multiple groups. Differences were considered statistically significant at *p* < 0.05.

## 3 Results

### 3.1 Identification of DEGs between POCD samples and controls

**Figure 1:**
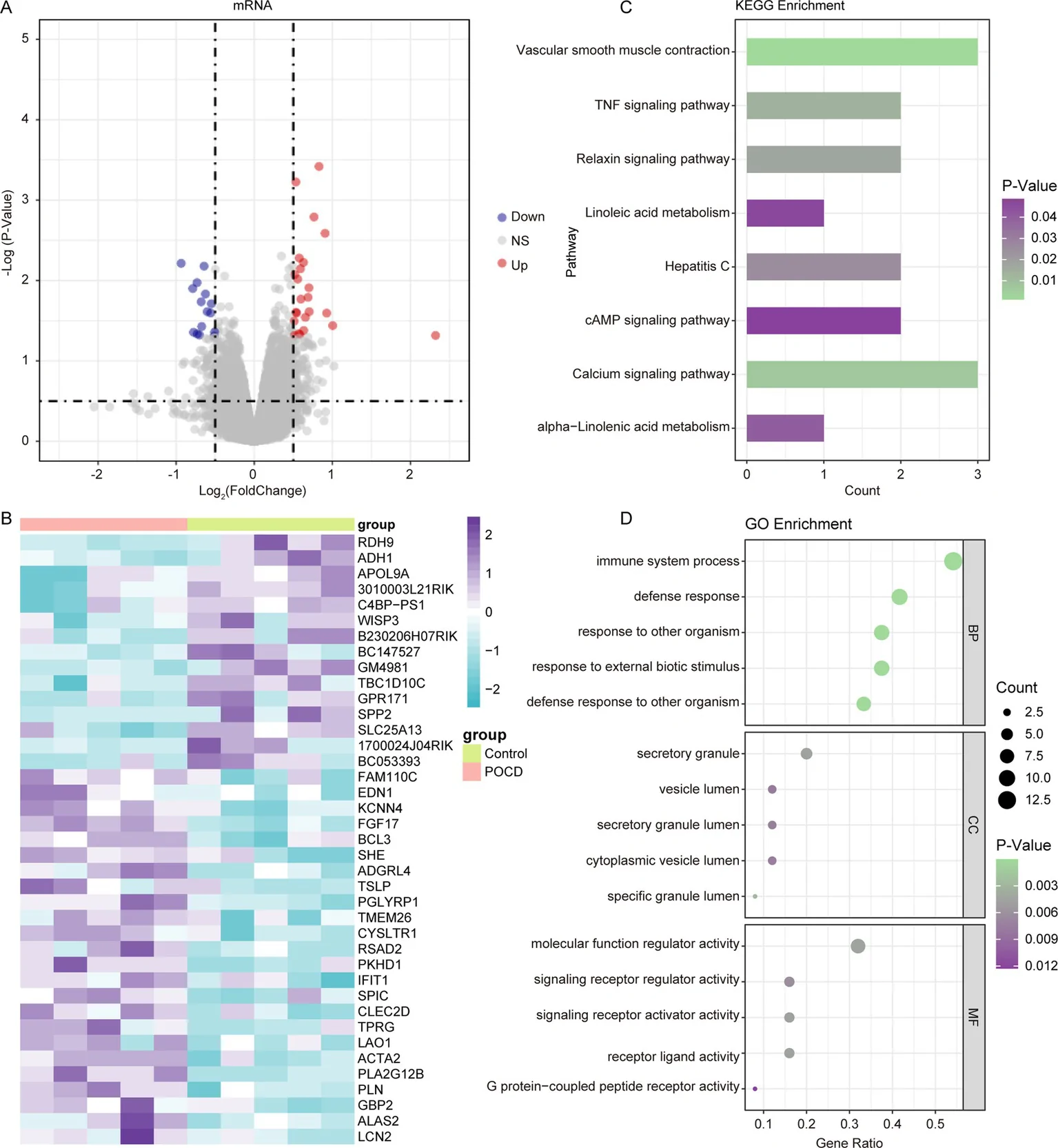
Identification of DEGs between POCD and control samples. **(A)** Volcano plot of DEGs between POCD and control groups. Red and blue dots represent significantly up- and down-regulated genes, respectively. **(B)** Heatmap showing the expression profiles of DEGs between POCD and control groups. **(C)** The bar chart illustrates the results of the KEGG enrichment analysis for DEGs. **(D)** The bubble chart illustrates the top five enriched GO terms in each category (BP, CC, and MF) for DEGs.

To identify DEGs between POCD and control groups, we performed differential expression analysis on the merged mRNA-seq datasets comprising five POCD samples and five control samples. The results showed that there were 39 DEGs, including 15 down-regulated and 24 up-regulated genes in the POCD group compared to controls (Figures 1A,B; Supplementary Table S3). KEGG and GO enrichment analyses were subsequently performed to explore the functional pathways of these DEGs. The KEGG enrichment analysis results showed that these genes were significantly enriched in eight pathways such as cAMP signaling pathway and TNF signaling pathway (Figure 1C; Supplementary Table S4). The results of GO enrichment analysis showed that these genes significantly participated in 380 GO-BP terms like immune system process, 22 GO-CC terms such as secretory granule, and 27 GO-MF terms such as molecular function regulator activity (Supplementary Table S4). The top five GO terms were shown in Figure 1D.

### 3.2 Screening of genes linked to programmed cell death, inflammation, immunity, and oxidative stress in POCD

**Figure 2:**
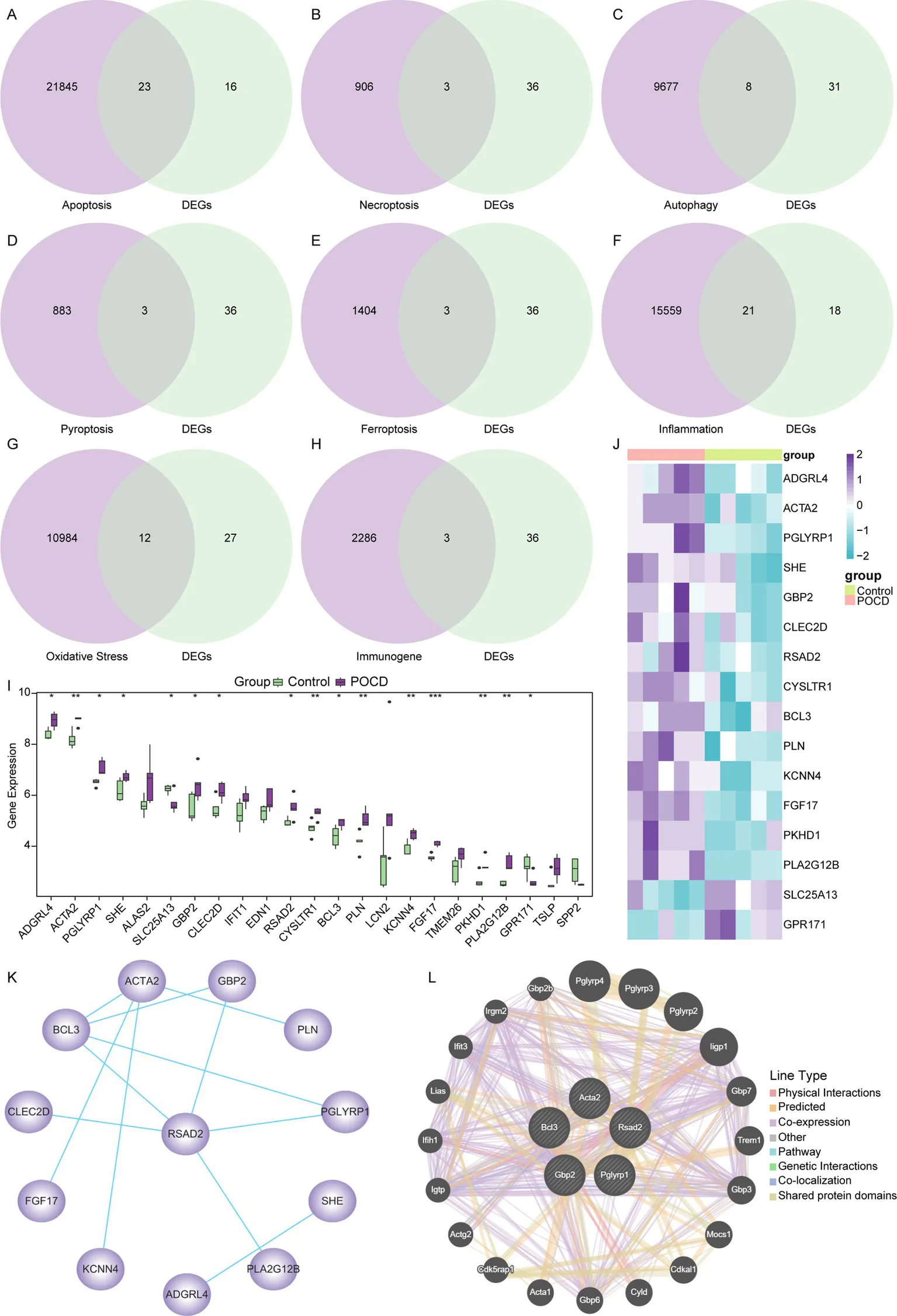
Identification of genes linked to programmed cell death, inflammation, immunity, and oxidative stress in POCD. **(A–H)** Venn diagrams illustrating overlaps between DEGs and gene sets related to **(A)** apoptosis, **(B)** necroptosis, **(C)** autophagy, **(D)** pyroptosis, **(E)** ferroptosis, **(F)** inflammation, **(G)** oxidative stress, and **(H)** immunogene. **(I)** Box plot comparing expression levels of the 23 overlapping genes identified from Venn analyses between POCD and control groups (* *p* < 0.05, **p < 0.01, ***p < 0.001). **(J)** Heatmap of 16 DEmRNAs expression levels in POCD and control groups. **(K)** PPI network of DEmRNAs. **(L)** Network interaction map of DEmRNAs based on GENEMANIA.

To identify genes associated with programmed cell death, inflammation, immunity, and oxidative stress in POCD, we intersected 39 DEGs with gene sets retrieved from the Genecards database (Supplementary Table S5). This analysis revealed eight relevant gene sets, including apoptosis, necroptosis, autophagy, pyroptosis, ferroptosis, inflammation, oxidative stress, and immunogene, which intersected with DEGs (Figures 2A–H), resulting in a total of 23 genes (Supplementary Table S5). Subsequently, we examined the expression patterns of these 23 genes in the POCD and control samples. Our findings revealed that 16 genes exhibited statistically significant alterations in their expression levels, designated as DEmRNAs, between the POCD and control groups. Specifically, compared to the control group, 14 DEmRNAs, such as ADGRL4, were significantly up-regulated, whereas two DEmRNAs, including SLC25A13 and GPR171, were significantly down-regulated in the POCD group (Figures 2I,J; Supplementary Table S5). To further elucidate the complex interactions among these DEmRNAs, we constructed a PPI network. The results demonstrated a cross-regulatory relationship among RSAD2, BCL3, ACTA2, PGLYRP1, and GBP2 proteins, suggesting their potential roles in POCD (Figures 2K,L).

### 3.3 Identification of DEmiRNAs and DEcircRNAs between POCD samples and controls

**Figure 3:**
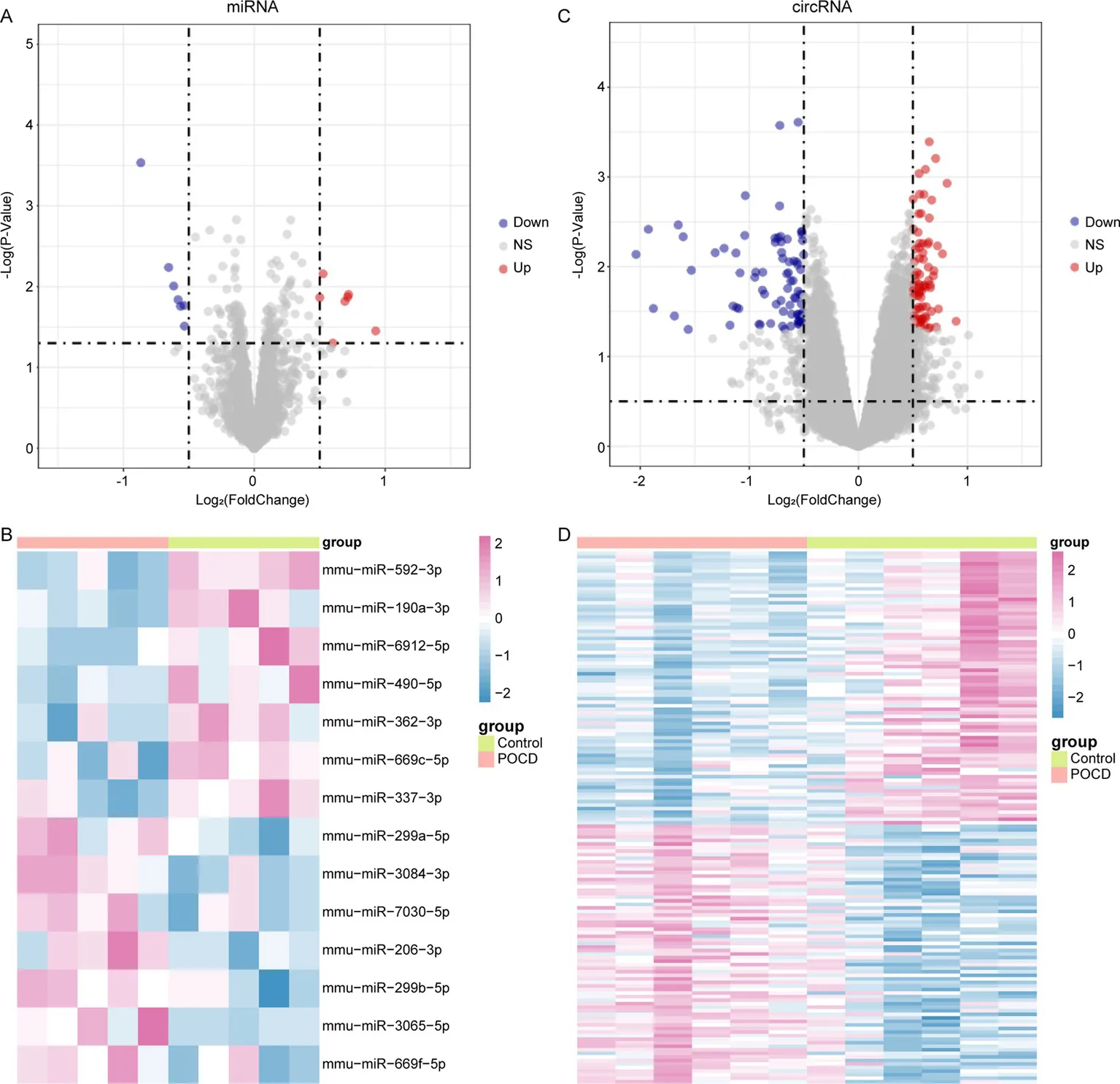
Identification of DEmiRNAs and DEcircRNAs between POCD samples and controls. **(A)** Volcano plot of DEmiRNAs between POCD and control groups. Red and blue dots represent significantly up- and down-regulated miRNAs, respectively. **(B)** Heatmap showing the expression profiles of DEmiRNAs between POCD and control groups. **(C)** Volcano plot of DEcircRNAs between POCD and control groups. Red and blue dots represent significantly up- and down-regulated circRNAs, respectively. **(D)** Heatmap showing the expression profiles of DEcircRNAs between POCD and control groups.

To screen DEmiRNAs and DEcircRNAs between POCD samples and controls, we performed differential expression analyses on the respective miRNA and circRNA expression profile data. The DEmiRNA analysis revealed 14 DEmiRNAs, including seven that were down-regulated and seven that were up-regulated in the POCD group compared to the control group (Figures 3A,B; Supplementary Table S3). Additionally, the DEcircRNA analysis identified 150 DEcircRNAs, including 77 that were down-regulated and 73 that were up-regulated in the POCD group compared to the control group (Figures 3C,D; Supplementary Table S3).

### 3.4 Prediction of the circRNA-miRNA-mRNA network

**Figure 4:**
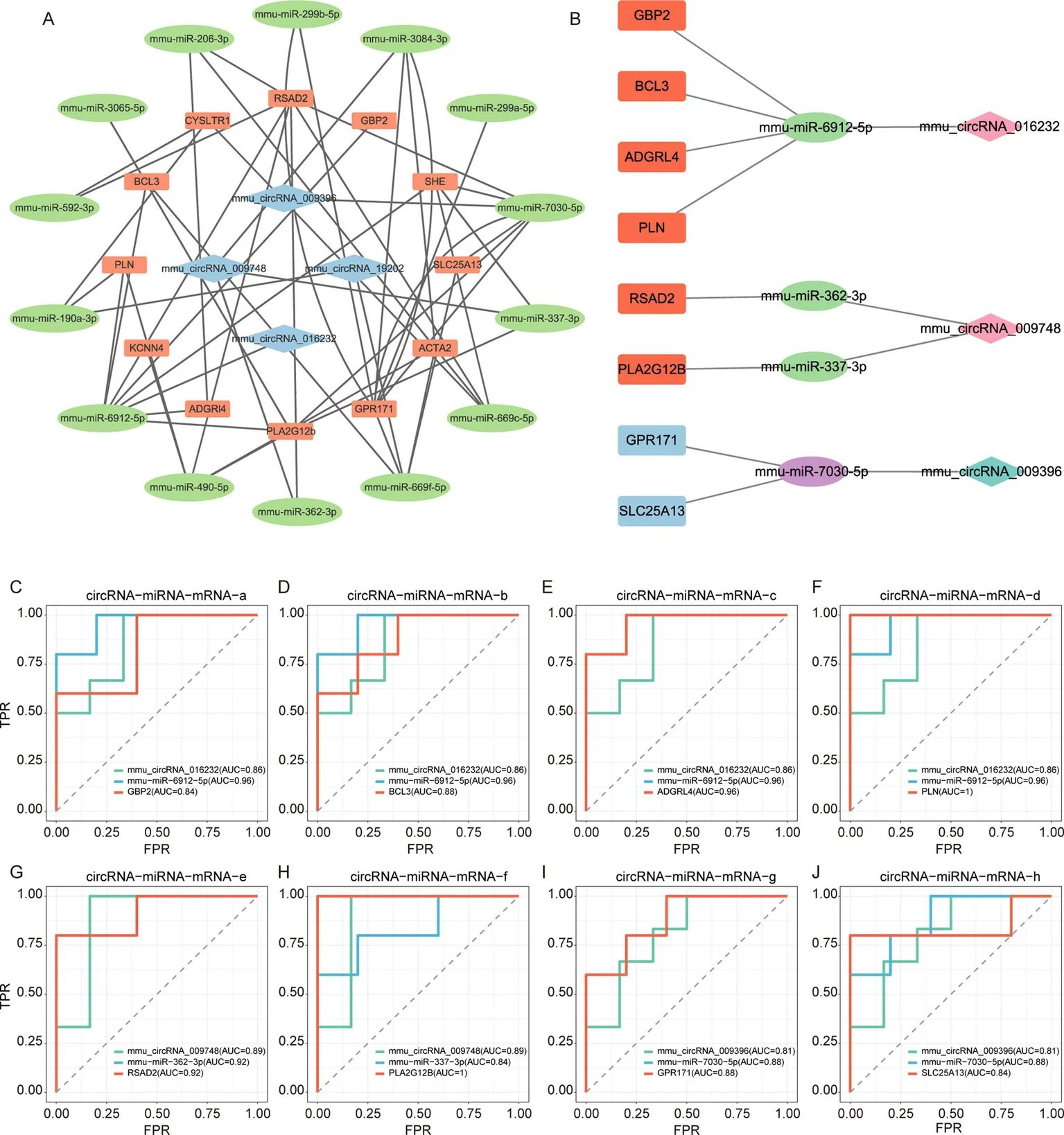
Prediction of the circRNA-miRNA-mRNA network. **(A)** A circRNA-miRNA-mRNA network of four circRNAs rhombus, 14 miRNAs (ovals), and 12 mRNAs (rectangles). **(B)** Three key sub-networks extracted from **(A)**, each centered on a specific circRNA and involving four miRNAs and eight mRNAs in total. Pink and cyan rhombus represented up-regulated circRNAs and down-regulated circRNAs in the POCD group compared to control group, respectively. Green and purple ovals represented down-regulated miRNAs and up-regulated miRNAs in the POCD group compared to control group, respectively. Red and blue rectangles represented up-regulated mRNAs and down-regulated mRNAs in the POCD group compared to control group, respectively. **(C–J)** Receiver operating characteristics (ROC) curves of each circRNA-miRNA-mRNA axis for discriminating POCD patients from controls.

To gain a deeper insight into the functional roles of DEmRNAs, DEmiRNAs, and DEcircRNAs in POCD, we predicted a circRNA-miRNA-mRNA network. This intricate network comprised 30 nodes, including 4 DEcircRNAs, 14 DEmiRNAs, and 12 DEmRNAs (Figure 4A). Based on the expression levels of these nodes, we identified three sub-networks. The first two subnetworks featured “up-regulated circRNAs” linked to “down-regulated miRNA” and “up-regulated mRNA” networks, centered on mmu_circRNA_016232 and mmu_circRNA_009748, respectively. The third sub-network consisted of a “down-regulated circRNA” linked to “up-regulated miRNA” and “down-regulated mRNA,” with mmu_circRNA_009396 as the central node. In total, the sub-networks identified three circRNAs (mmu_circRNA_016232, mmu_circRNA_009748, mmu_circRNA_009396), four miRNAs (mmu-miR-6912-5p, mmu-miR-362-3p, mmu-miR-337-3p, mmu-miR-7030-5p), and eight mRNAs (GBP2, BCL3, ADGRL4, PLN, RSAD2, PLA2G12B, SLC25A13, and GPR171) (Figure 4B). To assess the diagnostic potential of these circRNA-miRNA-mRNA pathways in POCD, we performed ROC analysis for each circRNA-miRNA-mRNA axis. The results revealed that the AUC value of all pathways exceeded 0.8 (Figures 4C–J), indicating their strong discriminatory capacity between POCD patients and healthy controls. The eight genes identified within these sub-networks, including *GBP2*, *BCL3*, *ADGRL4*, *PLN*, *RSAD2*, *PLA2G12B*, *SLC25A13*, and *GPR171*, emerged as potential hub genes for POCD.

**Figure 5:**
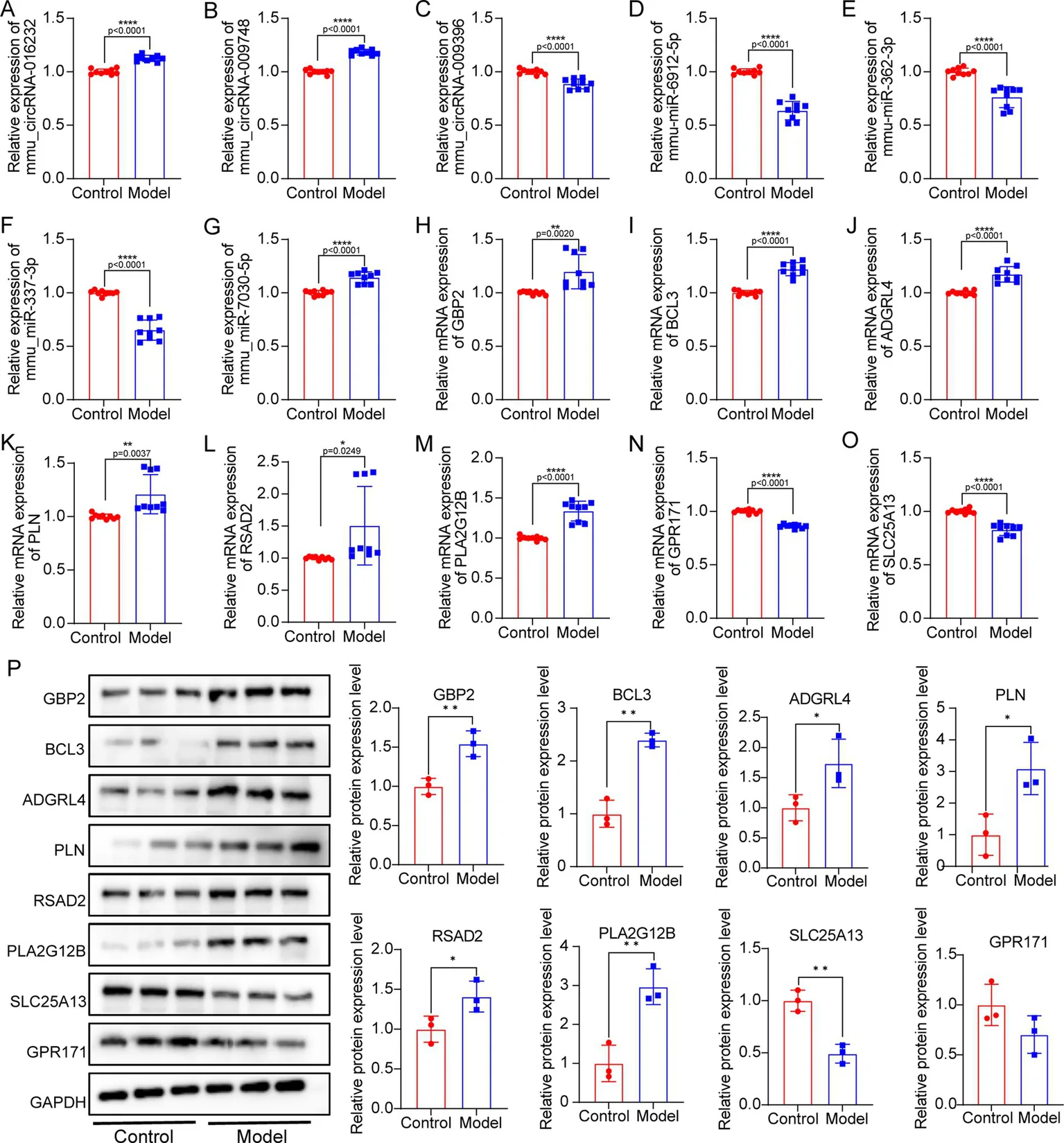
Expression levels of differentially expressed circRNAs, miRNAs, and mRNAs in hippocampal tissues of POCD mice. **(A–C)** RT-qPCR was conducted to determine the levels of three circRNAs (mmu_circRNA_016232, mmu_circRNA_009748, mmu_circRNA_009396) in the hippocampal tissues from control and POCD mice. **(D–G)** RT-qPCR was conducted to determine the levels of five miRNAs (mmu-miR-6912-5p, mmu-miR-362-3p, mmu-miR-337-3p, mmu-miR-7030-5p) in the hippocampal tissues from control and POCD mice. **(H–O)** RT-qPCR was conducted to determine the levels of eight mRNAs (GBP2, BCL3, ADGRL4, PLN, RSAD2, PLA2G12B, GPR171, and SLC25A13) in the hippocampal tissues from control and POCD mice. **(P)** Western blot was employed to determine the protein levels of eight genes (GBP2, BCL3, ADGRL4, PLN, RSAD2, PLA2G12B, GPR171, and SLC25A13) in the hippocampal tissues from control and POCD mice. *P < 0.05, **P < 0.01, ****p < 0.0001.

Furthermore, RT-qPCR was conducted to validate the expression levels of miRNAs and circRNAs in POCD mice. As shown in Figures 5A–G, the levels of mmu_circRNA_016232, mmu_circRNA_009748 and mmu-miR-7030-5p were notably elevated, and the levels of mmu_circRNA_009396, mmu-miR-6912-5p, mmu-miR-362-3p and mmu-miR-337-3p were significantly reduced in the hippocampal tissues of POCD mice compared to the control group. Moreover, RT-qPCR and western blot were employed to determine the expression levels of eight genes in POCD mice. As shown in Figures 5H–P, the mRNA and protein levels of GBP2, BCL3, ADGRL4, PLN, RSAD2, and PLA2G12B were remarkably increased, and the levels of SLC25A13 and GPR171 were decreased in the hippocampal tissues of POCD mice compared to the control group. These findings align with the results obtained from the bioinformatics analysis.

### 3.5 CircRNA_009748 positively regulates PLA2G12B mRNA expression by sponging miR-337-3p

Next, we performed luciferase reporter assays in HT22 cells to validate the interactions between mmu_circ_0001838 (mmu_circRNA_009748) and mmu-miR-337-3p, as well as between mmu-miR-337-3p and PLA2G12B mRNA. Overexpression of mmu-miR-337-3p markedly decreased the luciferase activity of reporters containing the wild-type mmu_circ_0001838 or containing cDNA to the 3’ UTR of PLA2G12B mRNA, whereas no effect was observed with the mutant mmu_circ_0001838 or mutant 3’ UTR cDNA (Supplementary Figures S1A,B). These results confirm direct binding between mmu_circ_0001838 and mmu-miR-337-3p, and between mmu-miR-337-3p and PLA2G12B mRNA. Furthermore, to investigate the regulatory relationship among mmu_circRNA_009748, mmu_miR-337-3p, and PLA2G12B mRNA, we first evaluated circRNA_009748 overexpression and circRNA_009748 siRNA knockdown efficiencies in HT22 cells. As shown in Supplementary Figure S1C, circRNA_009748-OE markedly elevated the expression of mmu-circRNA_009748 in HT22 cells. Conversely, specific siRNAs targeting mmu-circRNA_009748 effectively suppressed its expression, with mmu-circRNA_009748 siRNA2 exhibiting the most robust knockdown efficiency (Supplementary Figure S1D). Meanwhile, overexpression of mmu-circRNA_009748 significantly downregulated mmu-miR-337-3p in HT22 cells, whereas mmu-circRNA_009748 siRNA2 markedly upregulated its expression (Supplementary Figure S1E). Additionally, we also investigated the regulatory role of miR-337-3p on the downstream target PLA2G12B mRNA. As shown in Supplementary Figure S1F, mmu-miR-337-3p agomir and antagomir effectively upregulated and downregulated the expression of mmu-miR-337-3p in HT22 cells, respectively. Notably, mmu-miR-337-3p agomir-mediated overexpression significantly suppressed PLA2G12B mRNA expression, whereas inhibition of mmu-miR-337-3p by its antagomir markedly increased PLA2G12B mRNA expression (Supplementary Figure S1G). Rescue experiments were then performed to confirm this regulatory axis. As illustrated in Supplementary Figure S1H, knockdown of mmu-circRNA_009748 led to a reduction in PLA2G12B mRNA expression; however, co-transfection with mmu-miR-337-3p antagomir significantly rescued PLA2G12B mRNA levels. Collectively, these data indicate that circRNA_009748 functions as a miR-337-3p sponge, thereby positively regulating PLA2G12B mRNA expression.

### 3.6 Enrichment analysis of eight genes from the circRNA-miRNA-mRNA networks

**Figure 6:**
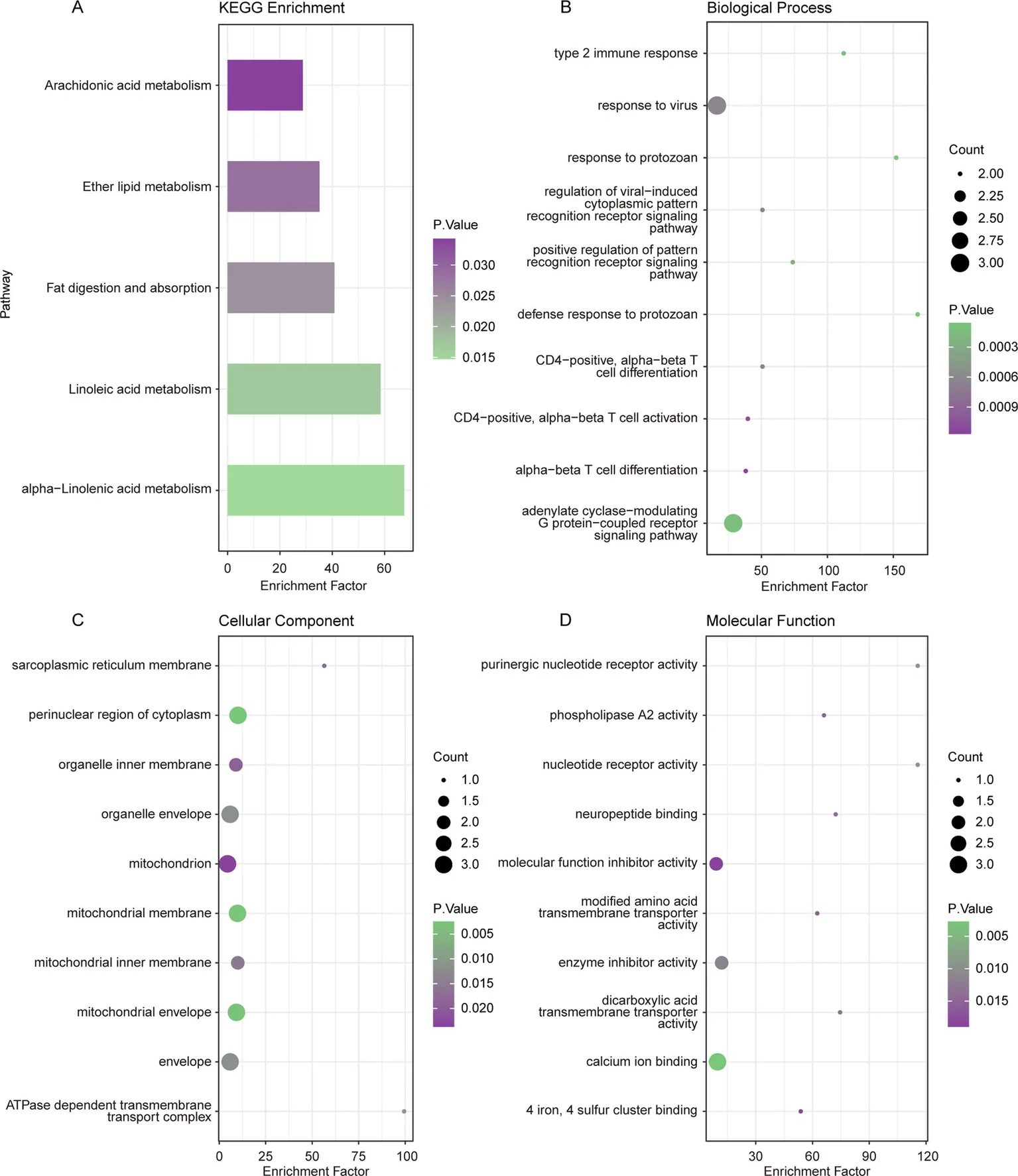
GO analysis and KEGG analysis of the eight genes in the circRNA-miRNA-mRNA networks in POCD. **(A)** KEGG pathway enrichment analysis of the eight genes in three key circRNA-miRNA-mRNA networks. **(B–D)** GO analysis of the eight genes in three key circRNA-miRNA-mRNA networks. The bubble chart illustrates the top ten enriched GO terms in each category (BP, CC, and MF) for these genes.

The circRNA-miRNA-mRNA regulatory network plays a crucial role in POCD. To gain insights into the underlying biological mechanisms of this network, we conducted KEGG and GO enrichment analyses on the eight genes identified within three sub-networks. The KEGG enrichment analysis revealed significant enrichment of these genes across five metabolic pathways such as alpha-linolenic acid metabolism, linoleic acid metabolism, and fat digestion and absorption (Figure 6A; Supplementary Table S6). Furthermore, the GO enrichment analysis indicated significant enrichment of 216 GO-BP terms, 15 GO-CC terms, and 24 GO-MF terms associated with stimulus response and immune response mechanisms (Figures 6B–D; Supplementary Table S6). These findings underscored the intricate involvement of the circRNA-miRNA-mRNA network in the regulatory landscape of POCD.

### 3.7 Exploration of the upstream transcription factors (TFs) of hub genes in POCD

**Figure 7:**
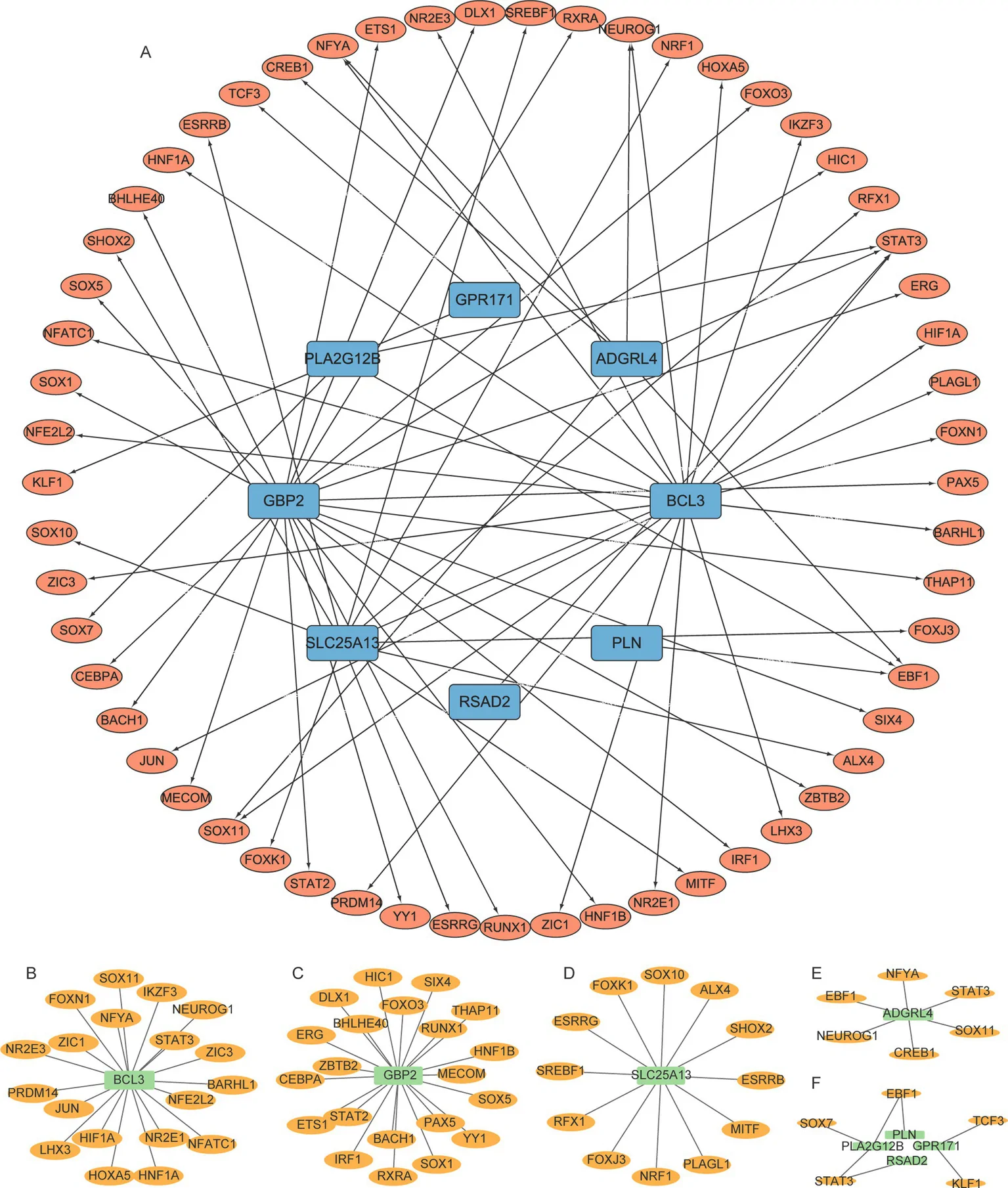
Exploration of the upstream TFs of eight genes in POCD. **(A)** Integrated network of the eight hub genes and predicted transcription factors (TFs) identified using the JASPAR database. **(B–F)** Sub-networks highlighting individual hub genes: **(B)** *BCL3*, **(C)** *GBP2*, **(D)** *SLC25A13*, **(E)** *ADGRL4*, and **(F)** the remaining four hub genes (*PLN*, *PLA2G12B*, *RSAD2*, and *GRP171*). Hub genes are represented by rectangles; TFs are represented by ovals.

To investigate the potential upstream regulatory mechanisms of the eight hub genes, we focused on performing a correlation analysis between these genes and TFs. Specifically, we calculated the expression correlation between the eight genes in the circRNA–miRNA–mRNA networks and 2,765 TFs (Supplementary Table S7). Based on the criteria of *p* < 0.05 and correlation coefficient > 0.6, we identified 57 TFs that showed significant associations with the eight genes (Supplementary Table S7; Figure 7A). Notably, four genes, including *BCL3*, *GBP2*, *SLC25A13*, and *ADGRL4*, were regulated by more than five TFs (Figures 7B–E), whereas the remaining four genes, including *PLA2G12B*, *PLN*, *GPR171*, and *RSAD2,* were regulated by less than five TFs (Figure 7F).

Next, we utilized the online tool FIMO to predict the binding sites of these 57 TFs in the upstream promoter regions of the eight genes. Our analysis revealed potential TF binding sites in the promoter regions of five genes: *PLA2G12B*, *GPR171*, *GBP2*, *BCL3*, and *ADGRL4*. Specifically, we identified four regions with potential EBF1-binding sites in the promoter region of *PLA2G12B*, five IRF1-bound regions upstream of *GBP2*, two SOX11-bound regions upstream of *ADGRL4*, one TCF3-bound region upstream of *GPR171*, and one STAT3-bound region upstream of *BCL3* (Supplementary Table S8). To further validate the reliability of the above predictions, we utilized the publicly available Cistrome ChIP-seq database to examine the binding interactions between the five genes and their corresponding transcription factors. The results revealed significant binding peaks for each TF within the promoter regions of their respective target genes (Supplementary Figure S2). This confirms that EBF1, IRF1, SOX11, TCF3, and STAT3 likely function as upstream transcription factors for *PLA2G12B*, *GBP2*, *ADGRL4*, *GPR171*, and *BCL3*, respectively.

## 4 Discussion

POCD significantly affected the quality of life of patients, especially in the elderly population, which exhibits a lower tolerance to surgical stress (Lin et al., 2020). Elucidating the regulatory networks of gene expression was of paramount importance for understanding the pathological mechanisms of POCD. Within these networks, circRNAs can competitively inhibit the binding affinity of miRNAs to their mRNA targets (Meng et al., 2017). Qian X. et al. (2023) reported that circUBE3B promotes hippocampal neuron injury through the miR-326/MYD88 axis and may participate in the pathological process of sevoflurane-induced POCD. Shuai et al. (2023) demonstrated that inhibition of circ_0016760 mitigates damage to hippocampal neurons caused by sevoflurane through the regulation of miR-145 expression. Additionally, He et al. (2023) showed that the circ-Shank3/miR-140-3p/TLR4 axis is also involved in neuroinflammation and may play a role in POCD. Collectively, these findings indicate that circRNAs, as core components of the ceRNA network, are widely involved in neuroinflammation and cell apoptosis in POCD. However, systematic studies on the circRNA-miRNA-mRNA network in POCD remain limited. In this study, we used microarray data and conducted a bioinformatics analysis to identify the circRNA-miRNA-mRNA networks associated with inflammation, oxidative stress, and programmed cell death in POCD.

We conducted a differential expression analysis utilizing microarray data of POCD samples. As a result, we successfully predicted a circRNA-miRNA-mRNA network, including 4 DEcircRNAs, 14 DEmiRNAs and 12 DEmRNAs (Figure 4A). Based on the ceRNA theory, there is a positive relationship between circRNA and the expression of downstream target genes, whereas the expression levels of the target genes exhibit a negative correlation with miRNA levels (Wu et al., 2020; Li et al., 2021). In this study, we systematically predicted and partially validated circRNA-miRNA-mRNA regulatory networks associated with POCD using integrated bioinformatics and experimental approaches. Our network analysis identified three ceRNA axes centered on mmu_circRNA_016232, mmu_circRNA_009748, and mmu_circRNA_009396, respectively (Figure 4B). The in silico analysis showed that the mmu_circRNA_016232-centered network exhibited upregulation of circRNA_016232 and its target mRNAs (GBP2, BCL3, ADGRL4, PLN) alongside downregulation of mmu-miR-6912-5p. Similarly, the mmu_circRNA_009748-centered network was predicted to show upregulation of circRNA_009748 and its target mRNAs (RSAD2, PLA2G12B) with downregulation of mmu-miR-362-3p and mmu-miR-337-3p. In contrast, the mmu_circRNA_009396-centered network showed an opposite bioinformatically predicted pattern: downregulation of circRNA_009396 and its target mRNAs (SLC25A13, GPR171) with upregulation of mmu-miR-7030-5p. These antiparallel changes in expression levels are consistent with the ceRNA regulatory model.

Among these axes, we focused on the mmu_circRNA_009748-mmu_miR-337-3p-PLA2G12B mRNA pathway for functional validation. Using HT-22 cells, we demonstrated that circRNA_009748 overexpression significantly suppressed miR-337-3p expression, whereas circRNA_009748 knockdown increased miR-337-3p levels. Moreover, miR-337-3p agomir reduced PLA2G12B mRNA expression, while antagomir treatment elevated PLA2G12B mRNA expression. Rescue experiments further confirmed that circRNA_009748 regulates PLA2G12B mRNA specifically through miR-337-3p sponging. These findings provide direct evidence that circRNA_009748 functions as a miR-337-3p sponge, thereby positively regulating PLA2G12B mRNA expression in hippocampal neurons.

The eight hub genes identified in our networks (*GBP2*, *BCL3*, *ADGRL4*, *PLN*, *RSAD2*, *PLA2G12B*, *SLC25A13*, and *GPR171*) have been implicated in neuroinflammation, apoptosis, or metabolic regulation (Bao et al., 2025; Miao et al., 2017; Yin et al., 2023; Sheldon et al., 2021; MacLennan et al., 1998; Qi et al., 2025; Fu et al., 2023; Baskar et al., 2023; Kou et al., 2025). Specifically, *GBP2* and *BCL3* are extensively involved in neuroinflammation and apoptosis (Miao et al., 2017; Bao et al., 2025; Yin et al., 2023), with GBP2 upregulated in POCD patients (Bao et al., 2025) and BCL3 linked to microglial inflammation (Zhao et al., 2025). Meanwhile, ADGRL4 participates in wound repair and inflammatory responses (Sheldon et al., 2022), while PLN is involved in cellular calcium homeostasis (MacLennan et al., 1998). Additionally, RSAD2 and PLA2G12B may contribute to inflammation through NF-κB pathway activation and arachidonic acid metabolism, respectively (Qi et al., 2025; Fu et al., 2023). Similarly, SLC25A13 and GPR171 are also associated with metabolic regulation. SLC25A13 deficiency causes hyperammonemia—a metabolic disturbance that can lead to delirium and brain damage (Baskar et al., 2023; Haberle, 2024; Komatsu et al., 2023; Ow et al., 2025); while GPR171 deficiency disrupts lipid metabolism and promotes inflammatory responses (Ram et al., 2021; Kou et al., 2025). Collectively, these genes converge on common pathological pathways, including neuroinflammation, programmed cell death, and metabolic dysregulation, that are central to POCD pathogenesis, supporting their potential relevance as candidate targets for further investigation. However, the direct involvement of these genes in POCD pathogenesis warrants further investigation.

Several limitations should be acknowledged. While we have experimentally validated the circRNA_009748-miR-337-3p-PLA2G12B mRNA axis, the other predicted networks remain to be verified through similar functional studies in future investigations. Second, the mechanisms of circRNA dysregulation in POCD remain unknown. Given that DHX9 negatively regulates circRNA biogenesis by unwinding back-splicing-associated RNA duplexes (He et al., 2024; Wang X. et al., 2022), and that DHX9 is critical for hippocampal function (Yang et al., 2024), we hypothesize that anesthetic-induced DHX9 downregulation may upregulate specific circRNAs (e.g., mmu_circRNA_016232 and mmu_circRNA_009748) in POCD. This hypothetical mechanism requires validation in the future through experimental studies. Finally, among the eight hub genes identified in this study, only *GBP2* and *BCL3* have been preliminarily investigated in POCD (Bao et al., 2025; Zhao et al., 2025); the remaining six genes have not been previously studied in this context. Although our bioinformatic analysis revealed their differential expression in the hippocampus of POCD mice, their roles in POCD pathogenesis remain largely speculative and warrant future investigation.

## 5 Conclusion

We successfully predicted several POCD-associated circRNA-miRNA-mRNA networks. Our main novel findings include: (1) prediction of three ceRNA subnetworks linked to neuroinflammation or cell death; (2) identification of three previously unreported key circRNAs (mmu_circRNA_016232, mmu_circRNA_009748, and mmu_circRNA_009396); and (3) RT-qPCR validation of gene expression changes within the three subnetworks in the hippocampus of POCD mice.

Network analysis revealed eight core hub genes linked to immune regulation, inflammatory response, oxidative stress, cell death, and metabolism (*GBP2*, *BCL3*, *ADGRL4*, *PLN*, *RSAD2*, *PLA2G12B*, *SLC25A13*, and *GPR171*). Nevertheless, our research provides a predicted circRNA-miRNA-mRNA interaction network based on bioinformatic analysis and expression validation, and functional validation of these predicted axes in hippocampal neurons or *in vivo* remains to be completed.

## Data Availability

All data analyzed in this study were obtained from publicly available databases. No new raw data were generated in this study. The datasets analyzed are publicly accessible in the Gene Expression Omnibus (GEO) database under the following accession numbers: GSE115440, GSE215410, GSE95070, GSE165798 and GSE190880.

## Footnotes

1
www.ncbi.nlm.nih.gov/geo/

2
https://www.genecards.org/

3
https://string-db.org/

4
http://www.genemania.org

5
http://mirwalk.umm.uni-heidelberg.de/

6
https://mirtarbase.cuhk.edu.cn/

7
https://www.targetscan.org/vert_80/

8
https://mirdb.org/mining.html

9
https://rnasysu.com/encori/index.php

10
https://bis.zju.edu.cn/CircFunBase/

11
http://genome.ucsc.edu/

12
https://jaspar.genereg.net/

13
https://meme-suite.org/meme/tools/fimo

14
http://cistrome.org/db/#/

## Supplementary material

Supplementary Figure S1 Validation of the circRNA_009748/miR-337-3p/PLA2G12B regulatory axis in HT22 cells. **(A,B)** Dual-luciferase reporter assays in HT22 cells confirming the direct binding of mmu_circ_0001838 (mmu_circRNA_009748) to mmu-miR-337-3p, and of mmu-miR-337-3p to the 3' UTR of PLA2G12B mRNA. **(C,D)** RT-qPCR analysis of mmu-circRNA_009748 expression levels in HT22 cells transfected with circRNA_009748-OE plasmid **(C)** or specific siRNAs (siRNA1, siRNA2, and siRNA3) **(D)**. **(E)** RT-qPCR analysis of mmu-miR-337-3p expression in HT22 cells transfected with circRNA_009748-OE plasmid or mmu-circRNA_009748 siRNA2. **(F,G)** RT-qPCR analysis of mmu-miR-337-3p **(F)** and PLA2G12B mRNA (G) levels in HT22 cells transfected with miR-337-3p agomir or antagomir. **(H)** RT-qPCR analysis of PLA2G12B mRNA expression in HT22 cells transfected with circRNA_009748 siRNA2 or co-transfected with circRNA_009748 siRNA2 + miR-337-3p antagomir. **P* < 0.05, ***P* < 0.01, ****P* < 0.001, *****P*<0.0001.

Supplementary Figure S2 Validation of transcription factor binding to target gene promoters via Cistrome ChIP-seq database. **(A–E)** Binding peaks for each TF within the promoter regions of their respective target genes: **(A)** *PLA2G12B*, **(B)** *GBP2*, **(C)** *GRP171*, **(D)** *BCL3*, **(E)** *ADGRL4*. From top to bottom were genome chromosome locations, binding peaks, and gene positions. Arrows on the gene lines represented the direction of transcription.
